# Case report: Preliminary experience with organ-preserving treatment for locally advanced esophageal cancer in the immunotherapy era: a case series of four patients

**DOI:** 10.3389/fonc.2025.1700288

**Published:** 2025-12-11

**Authors:** Genrong Yang, Han Wang

**Affiliations:** Department of Thoracic Surgery, The First Affiliated Hospital of Henan University of Traditional Chinese Medicine, Zhengzhou, China

**Keywords:** immunotherapy, esophageal cancer, organ preservation, radiothearpy, chemtherapy

## Abstract

**Background:**

For resectable locally advanced esophageal cancer, a comprehensive treatment strategy primarily centered on surgery is commonly adopted in clinical practice. Although the minimally invasive approach has reduced surgical trauma, the altered postoperative physiological structure often leads to impaired digestive function, reflux, and malnutrition, which significantly impact patients’ quality of life. With the incorporation of immune checkpoint inhibitors, some patients achieve a pathological complete response(pCR) after neoadjuvant therapy. As a result, these patients may become ideal candidates for organ-preservation strategies.

**Case presentation:**

A retrospective analysis was conducted on four patients with locally advanced esophageal cancer who received chemoimmunotherapy with selective radiotherapy. Pathological response and recurrence-free survival (RFS) were assessed. All four patients achieved complete pathological response. Cases 1, 2, and 3 were followed for 42 months, 31 months, and 25 months, respectively, with no recurrence. Case 4 experienced recurrence after 18 months of follow-up. Reinitiated chemoimmunotherapy was ineffective, and the tumor showed slow progression on subsequent lines of therapy.

**Conclusion:**

Chemoimmunotherapy with selective radiotherapy may offer an organ-preserving opportunity for locally advanced esophageal cancer, with some patients achieving long-term recurrence-free survival. However, efficacy varies individually, and further optimization of treatment strategies is warranted.

## Introduction

The treatment landscape for esophageal cancer demonstrates a significant dichotomy. For early-stage disease, a well-established system of endoscopic mucosal resection has been developed, achieving both oncological cure and functional preservation (1, 2). For patients with inoperable advanced esophageal cancer, treatment modalities including chemoradiotherapy, immunotherapy, and nutritional support have led to a marked improvement in survival rates (3).In contrast, the management of resectable locally advanced esophageal cancer presents a wider spectrum of clinical choices. The selection of a treatment strategy is often influenced by the patient’s preference for surgery, physical condition, and socioeconomic factors. Provided that survival outcomes are not compromised, a treatment approach that preserves esophageal function and avoids or delays surgery would be acceptable to the majority of patients. Currently, there is considerable debate regarding organ-preservation strategies for locally advanced esophageal cancer. Our team has summarized the clinical data of four cases with locally advanced esophageal cancer who achieved favorable outcomes through chemoimmunotherapy, selectively combined with radiotherapy. This case series aims to contribute valuable insights and serve as a reference for the ongoing discussion on organ-preservation strategies in this clinical context.

## Case presentation

**Figure 1:**
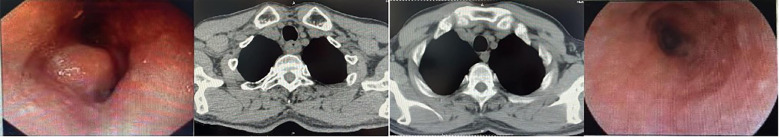
Comparison of gastroscopic images and CT before and after treatment.

Case 1. A 67-year-old male presented in February 2022 with a one-month history of dysphagia. Esophagogastroduodenoscopy (EGD) identified a neoplasm obstructing the esophageal lumen at 22 cm from the incisors, preventing passage of the endoscope (**Figure 1a**). Histopathological examination confirmed squamous cell carcinoma with a PD-L1 Combined Positive Score (CPS) ≥1. Contrast-enhanced chest computed tomography (CT) revealed a mass in the upper thoracic esophagus with several enlarged peri-tumoral lymph nodes (**Figure 1b**). The clinical stage was classified as cT3N1M0, stage IIIb (American Joint Committee on Cancer [AJCC] 8th edition). The patient had an Eastern Cooperative Oncology Group (ECOG) performance status of 0.History: Hypertension for over 20 years, well-controlled with amlodipine 5mg daily.30-pack-year smoker, non-drinker. No significant family history of genetic diseases or malignancies. He received two cycles of combination therapy with paclitaxel (135 mg/m², day 1), nedaplatin (80 mg/m², day 1), and camrelizumab (200 mg, day 1) administered every 3 weeks. Following this induction treatment, his dysphagia significantly improved. A follow-up chest CT demonstrated marked tumor regression (**Figure 1c**). After completing four cycles of this regimen, a repeat EGD showed slightly rough mucosa at 23–25 cm from the incisors, with clear vascular patterns and a patent lumen (**Figure 1d**). Biopsies confirmed no residual carcinoma, indicating a pathological complete response. Subsequent chest CT indicated near-normalization of the esophageal wall thickness. During two subsequent cycles of maintenance camrelizumab, the patient developed hypothyroidism, managed with oral levothyroxine. The patient declined further maintenance therapy and has been on regular surveillance. As of August 2025, he reports only occasional mild dysphagia with solid foods. Surveillance CT scans show no evidence of recurrence or metastasis, with a disease-free survival (DFS) exceeding 42 months.

**Figure 2:**
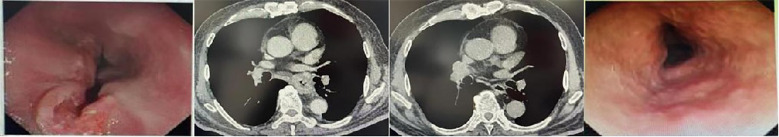
Comparison of gastroscopic images and CT before and after treatment.

Case 2. A 68-year-old male presented in December 2022 with a two-month history of progressive dysphagia. EGD revealed an irregular protruding lesion with central ulceration at 35 cm from the incisors (**Figure 2a**). Biopsy confirmed squamous cell carcinoma (PD-L1 CPS ≥1). Chest CT showed a tumor in the lower thoracic esophagus (**Figure 2b**). Clinical staging was cT3N0M0, stage IIa (ECOG 1).History: No history of hypertension or diabetes. Non-smoker, non-drinker. No significant family history of genetic diseases or malignancies. The patient initially declined treatment. One month later, with worsening dysphagia and back pain, he commenced therapy with paclitaxel (135 mg/m²), nedaplatin (80 mg/m²), and camrelizumab (200 mg) every 3 weeks. After two cycles, his symptoms improved, and CT confirmed significant tumor shrinkage (**Figure 2c**). He declined surgery and completed two additional cycles. A June 2023 EGD showed mildly irregular mucosa with somewhat indistinct vascularity at the tumor site (**Figure 2d**). Biopsies confirmed pCR. He then received 13 cycles of maintenance camrelizumab without immune-related adverse events (irAEs). Treatment was subsequently paused, and he remains on surveillance with no evidence of disease recurrence (DFS >31 months).

**Figure 3:**
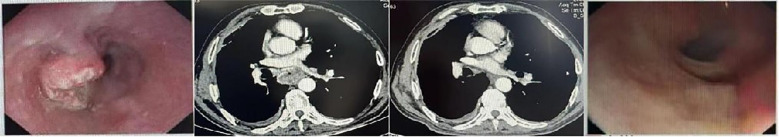
Comparison of gastroscopic images and CT before and after treatment.

Case 3. A 66-year-old male presented in July 2023 with a two-month history of dysphagia. EGD showed a neoplastic lesion with ulceration at 31–38 cm from the incisors (**Figure 3a**). Pathology confirmed squamous cell carcinoma (PD-L1 CPS ≥1). CT revealed a mid-to-lower esophageal mass with multiple enlarged periesophageal and upper mediastinal lymph nodes (**Figure 3b**). Staging was cT3N1-2M0, stage IIIb. His medical history included pleuritis, bilateral pleural thickening with calcification, and emphysema (ECOG 1).History: Type 2 diabetes for over 20 years, with suboptimal glycemic control on metformin and acarbose.80-pack-year smoker with occasional alcohol use. Significant family history of esophageal cancer in one brother. He was treated with two cycles of nab-paclitaxel (180 mg/m²), nedaplatin (80 mg/m²), and camrelizumab (200 mg) every 3 weeks, with symptomatic improvement. Follow-up CT showed marked tumor regression and reduced lymphadenopathy (**Figure 3c**). Given high surgical risk, he declined esophagectomy and received two additional cycles. A November 2023 EGD revealed a 5-cm longitudinal, clean-based ulcer on the left esophageal wall with visible vasculature and clear borders (**Figure 3d**). Biopsies confirmed pCR. He developed Grade 2 reactive cutaneous capillary endothelial proliferation (RCCEP) during treatment and declined maintenance therapy. He remains disease-free on surveillance (DFS >25 months).

**Figure 4:**
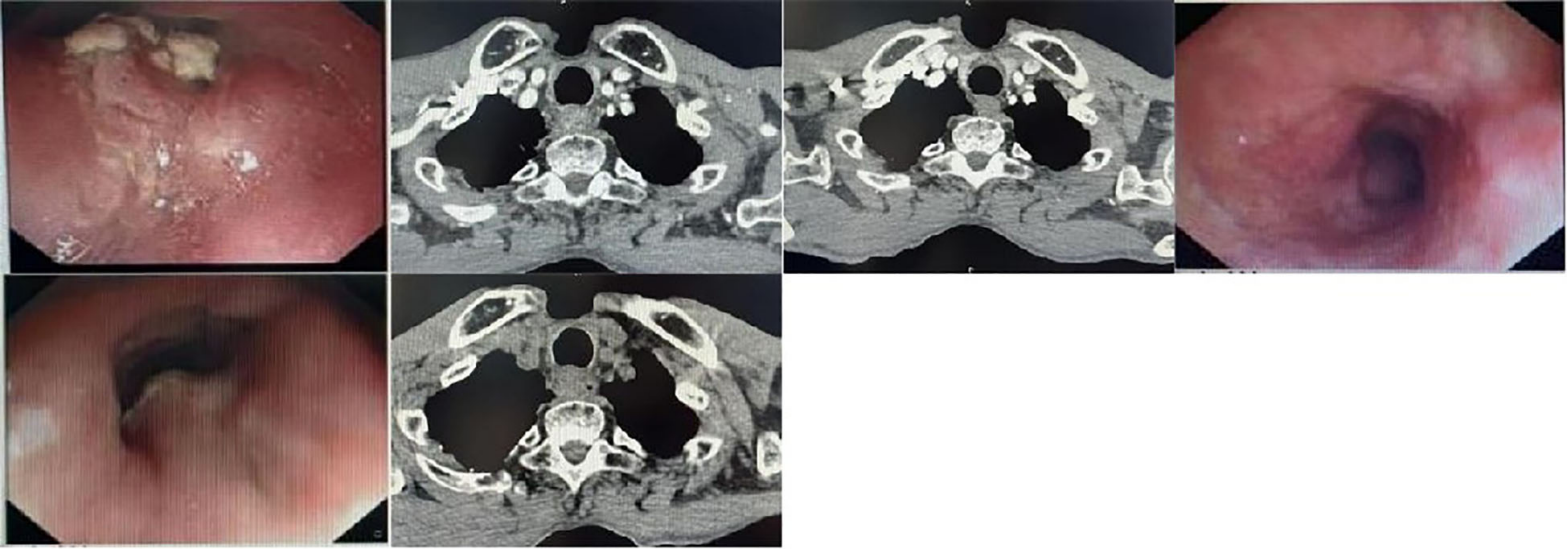
Comparative gastroscopic images and CT at pre-treatment, post-treatment, and recurrence timepoints.

Case 4. A 72-year-old male presented in August 2023 with a four-month history of dysphagia. EGD showed an irregular lesion on the left esophageal wall at 18–21 cm from the incisors (**Figure 4a**). Biopsy confirmed squamous cell carcinoma (PD-L1 CPS ≥10). CT revealed wall thickening and luminal narrowing in the upper esophagus with heterogeneous enhancement (**Figure 4b**). Staging was cT3N0M0, stage IIa (ECOG 1).History: No history of hypertension or diabetes. Non-smoker, non-drinker. No significant family history of genetic diseases or malignancies. He received one cycle of nab-paclitaxel (180 mg/m²), nedaplatin (80 mg/m²), and camrelizumab (200 mg). After symptomatic improvement, he received sequential radiotherapy (50.4 Gy) followed by three additional cycles of chemoimmunotherapy. Follow-up CT and EGD confirmed pCR (**Figures 4c, d**). After one cycle of maintenance camrelizumab, he developed Grade 2 RCCEP and was switched to sintilimab (200 mg every 3 weeks) for 13 cycles. Treatment was paused after confirming a clinical complete response (cCR).In March 2025, dysphagia recurred and progressed. EGD showed a circumferential, foul-coated mass obstructing the lumen at 20 cm (**Figure 4e**). Biopsy confirmed recurrence. CT showed progressive wall thickening (**Figure 4f**). Progression-free survival (PFS) was 18 months. He received one cycle of nab-paclitaxel, nedaplatin, and sintilimab without improvement. CT confirmed progressive disease (PD). In May 2025, he was switched to fluorouracil (500 mg/m² CIV 24h, d1-5), calcium folinate (200 mg/m², d1-5), and nedaplatin (80 mg/m², d1) plus oral anlotinib (12 mg daily, 2 weeks on/1 week off). He experienced Grade 2–3 nausea and vomiting and declined further chemotherapy. He continues on anlotinib monotherapy, tolerating a liquid diet, with an overall survival (OS) exceeding 24 months.

## Discussion and conclusions

The optimal organ preservation strategy for locally advanced esophageal cancer remains debated, particularly concerning the balance between oncological cure and functional outcomes (4, 5). Emerging evidence, such as the SANO trial, indicates no significant difference in OS or DFS between active surveillance and surgery for patients achieving a clinical complete response (cCR) after chemoradiotherapy. However, the SANO study primarily enrolled European patients, whose tumor biology may differ from that of Asian populations, and did not incorporate immunotherapy (6). The 2024 Chinese Society of Clinical Oncology (CSCO) Guidelines for Esophageal Cancer conditionally recommend organ preservation (Category 3) for patients who achieve radiological complete response and negative deep biopsy after chemoradiation, emphasizing close follow-up and salvage surgery (7). Current neoadjuvant recommendations prioritize chemoradiation or chemotherapy (8), while integrating immunotherapy has improved pCR rates. The ESCORT-NEO1 trial demonstrated a pCR rate of 28.0% with neoadjuvant nab-paclitaxel, cisplatin, and camrelizumab, reflecting a relevant and effective strategy in current Chinese clinical practice (9).

In Cases 1-3, radiotherapy was omitted based on excellent response to chemoimmunotherapy alone and to avoid potential complications such as radiation esophagitis or pneumonitis. Active surveillance was chosen after multidisciplinary discussion, reserving radiotherapy for progression. The combination of immunotherapy and radiotherapy exerts synergistic antitumor effects through the following mechanisms: Radiation induces immunogenic cell death (ICD) in tumor cells, releasing tumor-specific antigens and activating T cells to initiate systemic antitumor immune responses; Ionizing radiation upregulates PD-L1 expression in the tumor microenvironment (TME), thereby remodeling the immunosuppressive landscape and enhancing immune activation; Blockade of PD-L1 receptors reverses T-cell exhaustion, maintains T-cell immune homeostasis, and consequently augments the abscopal effect during radiotherapy while improving radiosensitivity (10, 11). Patients achieving pCR or significant response may avoid or delay surgery, considerably improving quality of life.

Case 4 involved cervical ESCC, where definitive radiotherapy offers superior functional outcomes compared to surgery requiring laryngectomy. Although pCR was achieved after sequential chemoimmunotherapy and radiotherapy, the disease progressed at 18 months. The sequential approach was selected to mitigate potential toxicities from concurrent immuno-radiotherapy, such as esophagitis, pneumonitis, or myelosuppression. The optimal timing and dose of radiotherapy in combination with immunotherapy remain undefined and require further investigation. The NICE study reported increased pCR rates but also higher rates of Grade 3 pneumonitis with concurrent chemoradiation and immunotherapy, though these findings are limited by the trial’s small sample size and single-arm design (12). The necessity and timing of radiotherapy for patients achieving pCR/cCR after chemoimmunotherapy are controversial. Deferring radiotherapy in responders may reduce toxicity and preserve quality of life but necessitates rigorous monitoring.

The standard response assessment system includes endoscopy, imaging preferably positron emission tomography-CT (PET-CT), and endoscopic ultrasound with fine-needle aspiration (EUS-FNA). The preSINO study incorporated circulating tumor DNA (ctDNA) analysis to improve residual disease detection after neoadjuvant chemoradiotherapy (nCRT), supporting organ preservation strategies in Asian ESCC patients (13). However, widespread implementation is limited by cost, technical requirements, and reimbursement policies. More accessible methods, such as endoscopic biopsy and CT, remain commonly used in routine practice, as reflected in our cases. Nonetheless, preSINO validates the accuracy of this multimodal approach in a Chinese cohort, providing a foundation for future research.

Surgery remains the cornerstone of multimodal treatment for resectable locally advanced esophageal cancer. Although surgical techniques and perioperative management have improved—reducing morbidity and enhancing survival and quality of life—outcomes still lag behind those achieved with successful organ preservation. Balancing cure and function requires robust clinical evidence to optimize treatment strategies and refine response assessment. This study innovatively explores the potential of chemotherapy combined with immunotherapy as an organ-preserving treatment strategy for esophageal cancer, thereby avoiding the need for definitive concurrent chemoradiotherapy in selected patients and reducing the incidence of radiation-induced toxicities such as esophagitis and pneumonitis. However, this study has several limitations: It is a single-center study with a small sample size and a relatively short follow-up period. Additionally, Cases 1–3 did not receive concurrent radiotherapy, a decision based primarily on efficacy evaluation. Moreover, as our center primarily uses PD-1 inhibitors, the combination with radiotherapy may increase the risk of complications such as pneumonitis. The use of PD-L1 inhibitors at our center has been limited due to restrictions in health insurance coverage and patients’ financial constraints. This case series aims to contribute insights into the impact of immunotherapy on organ preservation and its interactions with radiotherapy. We believe immunotherapy holds significant potential to facilitate organ preservation in appropriately selected patients with locally advanced esophageal cancer.

## Data Availability

The original contributions presented in the study are included in the article/supplementary material. Further inquiries can be directed to the corresponding author/s.
